# Refractory hemophagocytic lymphohistiocytosis secondary to polatuzumab vedotin plus rituximab and bendamustine

**DOI:** 10.1007/s00277-023-05598-4

**Published:** 2023-12-29

**Authors:** Kebing Lv, Fancong Kong, Min Yu, Yulan Zhou, Fei Li

**Affiliations:** 1 Center of Hematology, The First Affiliated Hospital of Nanchang University, No. 17 Yongwai StreetDonghu District, Nanchang, 330006 Jiangx China; 2 Jiangxi Clinical Research Center for Hematologic Disease, Nanchang, China; 3 Institute of Lymphoma and Myeloma, Nanchang University, Nanchang, China

## Dear Editor,

Immune and antibody drug–related hemophagocytic lymphohistiocytosis (HLH) is recognized as a rare adverse event (AE) with low incidence of 0.03 to approximately 0.4% but potentially lethal, with high mortality rate of up to 50% in some cases [1].

Polatuzumab vedotin (Pola) is a new type of antibody drug conjugate (ADC), and Pola-BR (pola plus rituximab and bendamustine) has been rapidly approved by the Food and Drug Administration (FDA) in June 2019 for R/R DLBCL [2, 3]. Here, we describe a patient who was on the treatment of Pola-BR and developed HLH.

A 66-year-old woman was diagnosed with stage IVB diffuse large B-cell lymphoma (DLBCL), accompanied by multiple lymphadenopathy and no bone marrow infiltration. The lymphoma remained uncontrolled after the first-line treatment with rituximab, cyclophosphamide, doxorubicin, vincristine, and prednisolone (R-CHOP); then rituximab plus gemcitabine, cisplatin, and dexamethasone (R-GDP), PD-1/Bcl-2/Btk inhibitor; and other multi-line treatments. The patient was then enrolled in the “Compassionate Use Program (CUP)” of Pola-BR and signed written informed consent (Ethics number: 2019164).

**Fig. 1:**
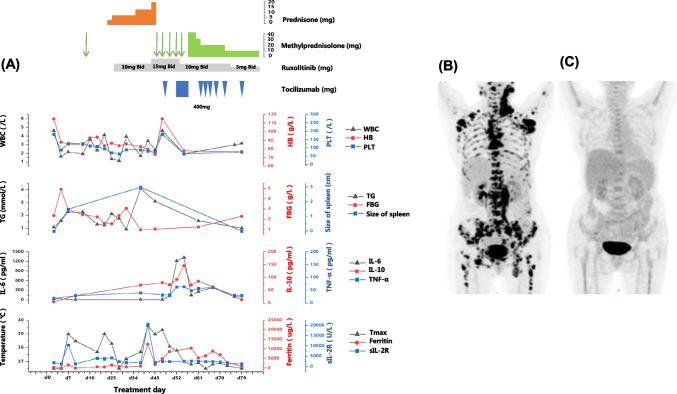
**A** Clinical and laboratory parameters relative to timing of Pola + BR and tocilizumab. On the 7th day of the Pola + BR treatment, the patient caught fever and CRS, with hyperferritinemia, increased sIL-2R, cytopenias, hypertriglyceridemia, and splenomegaly. On the 38th day of Pola + BR treatment, various examination indicators continued to deteriorate, and the patient was diagnosed with HLH and refractory to glucocorticoids and ruxolitinib. These improved significantly after continuous infusion of tocilizumab combined with gradual reduction, and HLH was assessed as PR after 4 weeks. **B** 2-[18F]fluoro-2-deoxy-d-glucose PET image of the patient at admission. Multiple lymph node enlargement in bilateral neck, bilateral axilla, mediastinum, retroperitoneal space, mesentery, and bilateral inguinal region (SUVmax 15.4). Diffuse mild bone destruction of the skull, vertebrae and accessories, ribs, sternum, and proximal extremities (SUVmax 12.2). **C** 2-[18F]fluoro-2-deoxy-d-glucose PET images of the patient after 4 courses of Pola + BR. Loss of tracer uptake indicates CR of the lymphoma

We plan to schedule patients to receive 6 cycles of the Pola + BR regimen, each with an interval of 21 days. On the 7th day of the Pola-BR, the patient caught a fever (between 38 and 39 ℃) and cytokine release syndrome (CRS) grade 1. Inflammation-related indicators including procalcitonin, 1,3-β-D-glucan test (G) and Galactomannan test (GM) were normal, and there were no signs of pneumonia on lung CT. Because of neutropenia, we use prednisone and ruxolitinib to prevent HLH and give empirical antimicrobial treatments such as Sulperazon, biapenem, and tigecycline. The patient still had recurrent fever. On day 38 of receiving Pola + BR, laboratory investigation revealed pancytopenia with a hemoglobin (Hb) of 69 g/L, white blood cell count of 1.43 × 10^9^/L (WBC) and platelets of 38 × 10^9^/L, Further work-up revealed high ferritin of 12,491 µg/L, fibrinogen (FBG) 1.0 g/L, increased soluble IL-2R (sIL-2R) level of 20,699 U/mL, and triglyceride (TG) levels of 4.1 mmol/L. And physical examination were splenomegaly. No relevant etiological evidence was found in the examination results: no increase in inflammation indicators, the lung CT was normal, and anti-infective therapy was ineffective; after Pola + BR treatment, the lymph nodes shrank, the LDH decreased, and there was no bone marrow involvement. So the inducements of tumor and infection were excluded. She met seven of the eight diagnostic criteria of HLH-2004 and was determined to be refractory to glucocorticoids and ruxolitinib (Fig. 1A).

According to the previous experience in the treatment of immunotherapy-related CRS [4], we used a dose of IL-6R inhibitor tocilizumab (TZC) as 8 mg/kg. However, the patient’s body temperature decreased slightly and then continued to increase to 39 °C, and ferritin and cytokine levels are on the rise. Therefore, we continuously used TZC for 5 days and gradually reduced to drug withdrawal. The patient’s body temperature and HLH-related indicators returned to normal after 10 days. It has been 2 months since the patient first showed symptoms such as fever, elevated cytokines, and elevated ferritin. The HLH-related curative effect was evaluated as partial remission (PR) after 4 weeks, and there was no recurrence of HLH during subsequent Pola + BR treatment. Surprisingly, her lymphoma was controlled and achieved PETCT complete remission (CR) after four courses of Pola-BR (Fig. 1B and C).

To date, there are no report about the Pola-induced HLH. This case highlights the need to share these experiences and provides a new diagnostic idea for clinical patients with high fever, hemocytopenia, and elevated ferritin after the application of this protocol, which is conducive to the identification of this dangerous complication for timely treatment. At the same time, we demonstrate the exciting efficacy of TZC in Pola-related HLH treatment.

## Data Availability

The data generated in this study are available upon request from the corresponding author.
